# Yeast for virus research

**DOI:** 10.15698/mic2017.10.592

**Published:** 2017-09-18

**Authors:** Richard Yuqi Zhao

**Affiliations:** 1 Department of Pathology, Department of Microbiology and Immunology, Institute of Global Health, and Institute of Human Virology, University of Maryland School of Medicine, Baltimore, MD 21201, USA.

**Keywords:** Saccharomyces cerevisiae, Schizosaccharomyces pombe, virus-host interaction, viral replication, cell cycle regulation, programed cell death, genome-wide analysis, high throughput drug screening

## Abstract

Budding yeast (*Saccharomyces cerevisiae*) and fission yeast (*Schizosaccharomyces pombe*) are two popular model organisms for virus research. They are natural hosts for viruses as they carry their own indigenous viruses. Both yeasts have been used for studies of plant, animal and human viruses. Many positive sense (+) RNA viruses and some DNA viruses replicate with various levels in yeasts, thus allowing study of those viral activities during viral life cycle. Yeasts are single cell eukaryotic organisms. Hence, many of the fundamental cellular functions such as cell cycle regulation or programed cell death are highly conserved from yeasts to higher eukaryotes. Therefore, they are particularly suited to study the impact of those viral activities on related cellular activities during virus-host interactions. Yeasts present many unique advantages in virus research over high eukaryotes. Yeast cells are easy to maintain in the laboratory with relative short doubling time. They are non-biohazardous, genetically amendable with small genomes that permit genome-wide analysis of virologic and cellular functions. In this review, similarities and differences of these two yeasts are described. Studies of virologic activities such as viral translation, viral replication and genome-wide study of virus-cell interactions in yeasts are highlighted. Impacts of viral proteins on basic cellular functions such as cell cycle regulation and programed cell death are discussed. Potential applications of using yeasts as hosts to carry out functional analysis of small viral genome and to develop high throughput drug screening platform for the discovery of antiviral drugs are presented.

## INTRODUCTION

### The two prize winning yeasts

Yeasts belong to the kingdom of fungi. The two most commonly used yeasts for virus research are budding yeast (*Saccharomyces cerevisiae*) and fission yeast (*Schizosaccharomyces pombe*). Budding yeast reproduces itself through a “budding” cell division process, i.e., a smaller daughter cell (a small bud or bleb) is initially formed on the mother cell. During mitosis, half of the chromatids are separated into the daughter cell. The bud continues to grow until it separates from the mother cell, forming a new cell 1. In contrast, fission yeast is divided by binary fission that produces a daughter cell with equal size to the mother cell. Hence, it is referred to as “fission yeast”. The budding yeast is also known as the “baker yeast” or the “brewer’s yeast” because it is commonly used commercially for baking breads or making top-fermented beers such as ale. The species name of fission yeast “pombe” is derived from the Swahili word for beer (pombé). It has been used in Central Africa to make pombe beer that is similar to bottom-fermented beers such as lager. Both yeasts are classified in the phylum of *Ascomycota*, i.e*.*, a group of fungi that produce ascospores during meiosis. Thus they are also known as the sac (ascus) fungi. Same as multicellular eukaryotes, they have nuclei and other membrane-bound organelles such as mitochondria, Golgi apparatus and a network of membranous tubules within the cytoplasm known as endoplasmic reticulum (ER).

Yeasts are single cell eukaryotic organisms. Many of the fundamental cellular functions such as cell proliferation, cell cycle regulation, cellular transport, cell self-destruction of intracellular contents or programed cell death are highly conserved from yeasts to higher eukaryotes. Therefore, yeasts are in many ways good models to study some of those highly conserved cellular activities. Indeed, the study of both budding and fission yeasts in the past half century has contributed significantly to advance our knowledge in human biology, physiology and cancer biology. Those significant contributions to science are exemplified by the awarding of three Nobel Prizes to scientists who were working on yeast model systems within a time period of fifteen years. Specifically, the shared 2001 Nobel Prize in Physiology and Medicine to three scientists including yeast biologists Drs. Leland Hartwell and Paul Nurse was for their seminal discoveries concerning the control of the cell cycle by using budding and fission yeast models, respectively 2,3. Dr. P. Nurse used the fission yeast model system and discovered Cdc2, a human homologue of CDK1, a key regulator of all eukaryotic cell cycle. Dr. L. Hartwell used budding yeast model system and discovered the “start” gene that was found to have a central role in controlling the first step of each cell cycle. Dr. Hartwell also introduced the concept “checkpoint”, a cellular surveillance system that safeguards the integrity of the cell cycle. Nearly all of the major cell cycle regulators identified in budding yeast and fission yeast have found their counterparts in mammalian cells 4,5. Defects in cell cycle control cause dysregulation of cell division and proliferation that may lead to cancers. Therefore, those fundamental discoveries have a great impact on all aspects of human cell biology. The 2013 Nobel Prize in Physiology or Medicine honored three scientists including another yeast biologist Dr. Randy W. Schekman who has solved the mystery of how the cell organizes its transport system. In particular, Dr. Schekman discovered a set of *Sec* genes that encode key regulators of the secretory pathway, which regulates vesicle transport in the cell. The importance of using yeast as a model to study human cell biology was once again cemented by the 2016 Nobel Prize in Physiology and Medicine. It was awarded to a single scientist, Dr. Yoshinori Ohsumi, for his discoveries of mechanisms for autophagy. His groundbreaking studies illuminated by using budding yeast as a model on how cells governs this intracellular degradation pathway to balance the cellular live and death process in response to various genotoxic stresses including viral infections 6,7.

There are numerous advantages of using yeasts to study heterologous gene activities over higher eukaryotes. For example, yeast cells are easy to culture in the laboratory. They grow rapidly with a doubling time of 3-5 hours. Cells producing heterologous proteins can be manipulated with various sophisticated molecular, cellular and genetic approaches. Traditional yeast genetic methods could be used to examine the gene effect in yeast on the loss-of-function by gene deletion; or on the gain-of-function by integrating a special gene of interest into the yeast chromosome. Genetic traits such as dominant or recessive phenotype of an identified cellular protein could be tested directly through haploid or diploid stages of the yeast life cycle. Finally, an identified cellular factor could also be verified by functional complementation using yeast or other eukaryotic homologues in respective cells. In fact, many human proteins that are important to human biology or diseases such as cancer-associated proteins were first discovered by studying their homologs in yeasts. For reviews of related topics, see 8,9,10,11.

There are also many benefits of using yeasts as model systems to study viruses of higher eukaryotes such as plant, animal or human viruses. The main reason is because yeasts carry their own indigenous viruses. Both positive sense (+) double stranded RNA (dsRNA) viruses, (+) single stranded RNA (ssRNA) viruses and retrotransposon elements have been reported in yeasts and other fungi 12,13. For example, studies of yeast killer viruses have helped us to study cellular necrosis and apoptosis during virus-host interaction 14,15,16,17, and to understand potential cellular viral restriction factors toward viral infections 18,19. Since the integration process of yeast retrotransposons resembles in many ways retroviral integration, molecular studies of fission yeast Tf elements or budding yeast Ty elements provided insights into functions of retroviruses such as HIV or murine leukemia viruses 20,21,22.

As shown in Table 1, many (+) RNA viruses and some DNA viruses replicate, to various degrees, in yeasts. For example, the first report showing yeast as a host for the replication of a plant viral genome was from Brome mosaic virus (BMV), which is a member of the alphavirus-like superfamily of animal and plant positive strand RNA viruses 23. In this study, yeast expressing BMV RNA replication genes *1a* and *2a* supports RNA-dependent replication and transcription of BMV RNA3 derivatives, suggesting all cellular factors that are essential for BMV RNA replication and transcription must be present in the yeast. Price *et al.* 24 described the first viral genome replication of animal virus, Flock House Virus (FHV) and its *de novo* synthesis of infectious virions in the yeast *Saccharomyces cerevisiae*. Besides the RNA viruses, the genomes of multiple human papillomavirus (HPV) subtypes and bovine papillomavirus (BPV) type 1 can stably replicate in yeast in an E1 or E2-independent manner as nuclear plasmids 25,26. This HPV viral gene E1 is a helicase, and E2 is a transcriptional activator and plasmid maintenance factor. Both are known to contribute to the episomal replication of the viral genome 25. This might be the first report showing an entire human viral genome can replicate as an episomal plasmid in yeast, suggesting yeast has the necessary cellular factors to support HPV/BPV replications.

**TABLE 1:** High eukaryotic viruses that replicate in yeast *S. cerevisiae*. Note: Modified and updated based on 27. Note that ASBVd is a viroid not a virus per se.

**Family**	**Virus**	**Genome**	**Natural host**	**Measurement of viral replication in yeast**	**References**
**RNA viruses**					
Bromoviridae	Brome mosaic virus (BMV)	(+)ssRNA	Plants	Replication gene 1a, 2a-dependent and RNA-dependent transcription and replication of BMV RNA3 derivatives	23
Tombusviridae	Carnation Italian ringspot virus (CIRV)	(+)ssRNA	Plants	Transcription and replication of CIRV DI-72 RNA that are supported by simultaneous expression of two replicase proteins (p36 and p95) in a three-plasmid system	30
	Tomato bushy stunt virus (TBSV)	(+)ssRNA	Plants	Transcription and replication of TBSV DI-7 RNA that are supported by simultaneous expression of two replicase proteins (p33 and p92) in a three-plasmid system	31
	Cymbidium ringspot virus (CRV)	(+)ssRNA	Plants	Similar to CIRV and from the same lab	32
Nodaviridae	Flock House virus (FHV)	(+)ssRNA	Animals	FHV genome replication and transcription in FHV virion RNA-transfected yeast spheroplasts; plaque formation on *Drosophila* cell monolayers	24
	Nodamura virus (NoV)	(+)ssRNA	Animals (Mammals)	Similar to FHV	28
Avsunviroidae	Avocado sunblotch viroid (ASBVd)	ssRNA circular	Plants	Self-cleavage and replication of ASBVd RNA strands of both polarities	33
**DNA viruses**					
Papillomaviridae	Human papillomavirus (HPV)	dsDNA circular	Humans	Amount of HPV genome DNA using a *Dpn*I resistance assay	25
	Bovine papillomavirus (BPV)	dsDNA circular	Animals	Same as HPV	26
Geminiviridae	Mung bean yellow mosaic India virus (MBYMIV)	ssDNA circular	Plants	Yeast colony size, PCR and southern blot measurement of viral replicated MBYMIV plasmid DNA	29
Parvoviridae	Adeno-associated virus (AAV)	ssDNA circular	Animals and Humans	Similar to MBYMIV	34

Yeasts also have much smaller genomes than higher eukaryotes. Study of higher eukaryotic viruses that replicate in yeasts could aid study of core relationship between a viral function and cellular proteins, thus avoiding high complexity and redundancy of higher eukaryotic systems. In addition, genome-wide single gene deletion yeast strain libraries and/or genomic DNA or cDNA plasmid libraries are widely available for both yeasts. Cellular factors that are involved in viral DNA replication can thus be identified by exposing the viral replication apparatus to those genomic libraries. In this case, loss or reduction of the viral replication in the absence of a cellular protein would suggest requirement or involvement of this cellular protein in viral replication. Similarly, cellular viral restriction factors could potentially be uncovered by overproduction of a genomic or cDNA plasmid library in the viral replicating yeast cells. Finally, many of the experimental approaches used in yeasts are not readily achievable in mammalian cells. For example, multiple and permanent heterologous gene-producing yeast strains can be established and maintained in the laboratory that allow simultaneous and batch testing repeatedly, thus facilitating large-scale and functional characterization of genes of interest such as a small viral genome 35,36. Therefore, study of virus-cell interaction by taking advantage of the simplicity, biosafety and genetic amenability of yeasts can often reveal novel scientific findings that are not always easy to discover solely by relying on high eukaryotic systems.

### The budding yeast (*Saccharomyces cerevisiae*)

**Figure 1:**
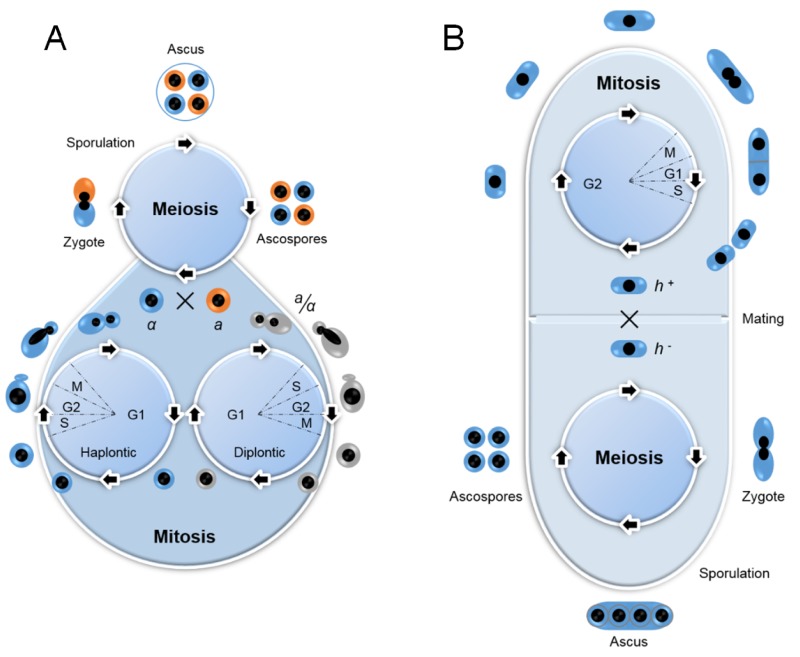
FIGURE 1: Life cycles of budding yeast (*Saccharomyces cerevisiae*) (A) and fission yeast (*Schizosaccharomyces pombe*) (B). Diagrams show yeasts have both asexual (vegetative) and sexual reproductive cycles, respectively. **(A)** Budding yeast is generally maintained in the laboratory through vegetative growth both as haplontic (haploid) and diplontic (diploid) cells during asexual life cycle by mitosis, which produce daughter cells by budding off of mother cells. Mitotic cell cycle has all of the typical eukaryotic cell cycle stages of G1, S, G2 and M phases, but it spends most of its cell cycling in G1 phase, which is similar to human cell cycle. Under stressful conditions, diploid cells undergo meiosis to form haploid spores by sporulation. Haploid cells of opposite mating types (a or α) can go on to mate (conjugate) and to reform diploid cells. **(B)** Fission yeast is normally present as haploid cells through mitosis. Cells are divided equally between daughter and mother cells. In contrast to budding yeast cell cycle, fission yeast spends most of its cell cycling in G2 phase. Its diploid form could be triggered by stress through mating of opposite mating type (*h^+^* or *h^-^*) during meiosis.

Budding yeast (*S. cerevisiae*) has sixteen chromosomes with a genome size of approximately 1.2 x 10^6^ base pairs (bps). It has about 5,700 protein-coding genes, with about 4.4% of them contain introns 37. *S. cerevisiae* cells are typically round to ovoid in shape with 5 - 10 μm in diameter. The daughter cells that are generated during cell division are generally smaller than mother cells (Fig. 1A). Unlike fission yeast, budding yeast’s cell wall contains both β-glucans and chitin. The optimum temperature for growth of *S. cerevisiae* is 30 - 35°C. For general experimental purposes, budding yeasts are usually grown in the complete yeast extract, peptone and dextrose (YPD) medium at 30°C without selection. Standard synthetic defined (SD) minimal medium is used to grow auxotrophic yeast cultures or select for yeast transformants containing plasmids. The selection media are generated by adding defined mixture of amino acids, vitamins and other components known as the drop-out supplements. A list of budding yeast selectable markers *HIS3*, *LEU2*, *TRP1* or *URA3* are used to select for the presence of a plasmid 38. Antibiotics such as hygromycin B and kanamycin can also be used as selectable markers 39,40.

Like other fungi, the life cycle of both budding and fission yeasts undergo asexual and sexual reproductive cycles (Fig. 1). They are generally maintained in the laboratory through vegetative growth by asexual reproduction 41. Unlike fission yeast, budding yeast reproduces both as haplontic (haploid) and diplontic (diploid) cells during asexual life cycle by mitosis (Fig. 1A). However, under high-stress conditions such as nutrient starvation, haploid cells will die; while diploid cells undergo meiosis to form haploid spores by sporulation 42,43. Haploid cells of opposite mating types (a or α) can go on to mate (conjugate) and to reform diploid cells 44. Budding yeast grows and divides through an asymmetric budding process. During mitosis, the daughter cell begins to form as a small bud on the tip of the mother cell. At metaphase, one set of sister chromatids moves into the bud. Continued growth of the bud eventually becomes a separated daughter cell. Budding yeasts have all of the typical eukaryotic cell cycle stages of G1, S, G2 and M (mitosis) phases, which can be recognized by DNA content, nuclear morphology and bud morphology. Budding yeast spends most of its cell cycling in G1 phase, which is similar to human cell cycle. Nearly all of the major cell cycle regulators identified in budding yeast have their counterparts in mammalian cells 4. Budding yeast has been used extensively as a model to study virus-host interactions and cellular restriction factors to viral infections 45,46. Genome-wide approaches have been used in budding yeast to study various virus-related activities including viral transcription, viral replication and virus-host interactions 46,47,48,49,50. General reviews on these topics are available 27,45,46,51,52.

### The fission yeast (*Schizosaccharomyces pombe*)

Fission yeast (*S. pombe*) has three chromosomes with the size of 5.7, 4.6, and 3.5 Mb (megabases), respectively 53. Its genome has approximately 1.4 x 10^6^ bps. It is estimated to have about 5,000 protein-coding genes. More than 90% of them contain introns 4,54. Subcellular locations of almost all fission yeast proteins are known 55. A typical fission yeast cell is rod-shaped that is normally 3 - 4 µm in diameter and 7 - 14 µm in length (Fig. 1B). Fission yeast cell is unique from other ascomycetous yeasts because its cell wall lacks chitin but deposits α-(1,3)-glucan or pseudonigeran in addition to the usual β-glucans. The optimal growth temperature for *S. pombe* cells in the laboratory is 30°C with a doubling time of 2 - 4 hours. The most commonly used growth medium with all of the necessary nutrients is the Standard Yeast Extract with Supplements (YES) medium, which is normally used to grow fission yeast cells without selection. The Edinburgh Minimal Medium (EMM) is typically used to select for the presence of a plasmid that carries a *LEU2* gene or *URA4* gene to compensate cellular gene defect in the *leu1-32* or *ura4-294* gene. In order to select for a *LEU2* or *URA4*-carrying plasmid, the EMM medium needs to be supplemented with leucine or uracil to complement the corresponding auxotrophic mutants of a yeast strain. Antibiotics such as cycloheximide and Zeocin have also been used to select for hygromycin and bleMX6 resistance in fission yeast cells 56,57.

Fission yeast is normally present as haplontic cells but its diploid form could be triggered during meiosis by mating when cells were subject to nutritional starvation (Fig. 1B, bottom) 11,58. Mating could take place between cells of two opposite mating types (heterothallic plus, *h^+^* or heterothallic minus, *h^-^*), or by self-cross of homothallic strains, e.g., *h^90^* that has both mating types 8,59. Fusion of the two cells results in the formation of diploid zygotes. Sporulation is followed immediately by meiosis to produce four round or oval haploid ascospores that are enclosed within an ascus. When appropriate nutrients are resumed to allow cells re-entering its asexual life cycle, the ascus wall will disintegrate, and ascospores will germinate and eventually divide to form haploid clones 8,60. The fission yeast cell maintains its shape by growing exclusively through its cell tips. After mitosis, cell division occurs by medial fission with the formation of a septum that cleaves the cell at its midpoint to produce two equal sized cells (Fig. 1B, top). Its specific length corresponds well with its growth phase in the cell cycle 61,62, which is similar to that of other eukaryotes, and includes the G1, S, G2, and M phases. However, *S. pombe* contains an extended G2 phase that can make up as much as 75% of its cell cycle 61. In addition, the fission yeast nuclear envelope remains intact throughout mitosis. Therefore, all transactions involving the chromosomes during this phase occur within the nucleus.

Fission yeast has been used extensively to study cell cycle regulation, DNA damage and repair as well as DNA replication. For example, like budding yeast, nearly all of the major cell cycle regulators identified in fission yeast have their counterparts in mammalian cells 4,5. It has also been used as a host system to study virus-host interactions including the effect of viral proteins on cell cycle regulation 63,64, gene expression, cell death and apoptosis 65,66,67. In addition, fission yeast has been used to carry out large-scale and functional characterization of small viral genomes such as human immunodeficiency virus type 1 (HIV-1) and Zika virus (ZIKV) 55,68,69,70. General reviews on some of the related topics have been published previously 11,22,64,71,72,73,74.

In summary, either fission yeast or budding yeast could be used as a reasonable model for the study of various viral activities and virus-host interactions. They often complement to each other in many ways. However, from the evolution perspective, these two yeasts diverged approximately 300 to 600 million years ago 75,76. Consequently, both yeasts have homologous genes with higher eukaryotes that they do not necessarily share with each other. Thus, it is important to know that there are sufficient functional differences between these two yeasts that sometimes could yield conflict results. For example, 96% of the fission yeast genes contain introns; whereas only 4% of the budding yeast gene carries introns 4,37,54. Similarly, fission yeast has RNAi machinery genes like those in vertebrates, but it is missing from budding yeast 77. Conversely, *S. cerevisiae* has well-developed peroxisomes, while *S. pombe* does not. Another example is that budding yeast has an extended G1 phase of the cell cycle. Thus, the G1-S transition is tightly controlled; whereas fission yeast spends most of its cell cycling time in the G2 phase of the cell cycle. Hence, the G2-M transition is under tight control. Therefore, the budding yeast might be a better choice to study cell cycle G1-S transition, whereas the fission yeast could serve a preferable role in the study of cell cycle G2-M regulation. Therefore, careful consideration has to be taken before choosing a model organism to study the viral genes of your interest. In the following sections, special emphasis is given to those studies that have generated significant discovery toward the understanding of a viral function or the virus-host interactions by using either yeast as a model system.

## INDIGENOUS YEAST VIRUSES

Yeasts have their own indigenous viruses. The budding yeast viruses include three families of dsRNA viruses (L-A, L-BC, and M), two families of ssRNA viruses (T and W), and five families of retrotransposons (Ty1 - Ty5) 12,13. The fission yeast includes retrovirus-like retrotransposons (Tf1 and Tf2) 78,79. Among those yeast viruses, the dsRNA and ssRNA viruses are infectious, as they are able to infect other healthy yeast cells, and to transmit themselves from cell to cell. As results of the yeast infection, some of those infectious yeast strains kill their receptive cells. Thus, they are also known as killer yeast. Historically, the final realization that the killer yeasts are actually associated with their own indigenous viruses took more than a century 80. Briefly, Louis Pasteur initially described the contribution of microbes to spoilage of beers in 1866 81. Horace Brown later linked the beer spoilage to *Saccharomyces* yeasts 82. Although it was known for quite a while that some yeast strains kill other yeast cells, the term “killer” yeast was first proposed by Wood and colleagues in 1968 83. The killer yeast strains secrete protein toxins (K1, K2, K28 and Klus) that are lethal to non-killer strains of the same or other species 84. Another synonyms term “zymocide” was also introduced in 1983 by Young *et al.* 85 to indicate that this killer yeast is only lethal to yeasts and not to bacteria or higher organism. Two dsRNA viruses were subsequently discovered in the killer yeast strains 86. Those two dsRNA viruses are the *S. cerevisiae* L-A virus (ScV-LA) and the M virus (ScV-M), respectively 87,88. However, not until 1987, El-Sherbeini and co-workers 12 demonstrated that yeast killer viruses are capable of extracellular transmissions. It was previously thought that killer yeast viruses transmit by cytoplasmic mixing during cell division, mating or other induced forms of cell fusion. Extracellular transmission was demonstrated by direction infection of K1 and K2 killer viral preparations to yeast spheroplasts, competent yeast cells by lithium acetate, or to mating cells 12. It is now known that the killing effect is achieved by the co-presence of ScV-LA and ScV-M viruses within the same yeast strain. Typically, these two viruses coevolve. Different ScV-M viruses could pair with a ScV-LA virus to form a unique yeast killer strain 87,88. The helper virus, ScV-LA, encodes the capsids for both viruses; and the K1, K2, K28, and Klus toxins are produced by different satellite ScV-M viruses 87,88. Therefore, the presence of a ScV-M dsRNA virus needs co-existence of a ScV-LA helper virus.

An interesting effect of the yeast killing effects is that, besides causing necrosis, they also induce apoptotic programed cell death by triggering caspase- or oxidative stress-mediated apoptosis 89. This apoptotic effects is seen during yeast viral infection of the receptive cells. Interestingly, however, the killer yeast cells themselves are immune to the toxic effects presumably due to intrinsic immunity 90. A set of yeast chromosomal gene products, SKI1, SKI2, SKI3, SKI6, SKI7, and SKI8, prevent the ScV-LA virus and its satellite ScV-M viral RNAs from harming their own cells 91,92. Thus, yeasts have been used to study cell apoptosis during virus-host interaction 14,15,16,17, and to understand potential cellular viral restriction factors toward viral infections 18,19.

Another intriguing fact is that the ScV-LA virus has two open reading frames (ORFs). The 5' *gag* gene encodes the major coat Gag protein, and the 3' *pol* gene encodes a multifunctional Pol protein that includes a RNA-dependent RNA polymerase (RdRP). The Gag-Pol fusion protein is produced by a minus-1 (-1) ribosomal frameshift during translation, a process that is identical to that used by higher retroviruses 92. Because of the functional similarities of the minus-1 translational ribosomal frameshift between yeast dsRNA viruses and that of higher eukaryotes 92, it provides a useful tool to delineate molecular actions of viral replication of higher eukaryotic retroviruses such as HIV, which is described in the next section.

## YEAST FOR THE STUDY OF VIRAL FUNCTIONS

A list of higher eukaryotic viruses that are known to replicate in yeast *S. cerevisiae* is included in Table 1. Those viruses include plant, animal and human viruses. Both RNA viruses and DNA viruses, including some of those pathologically important human viruses such as HPV, were found to replicate, in various degrees, in yeast. Those (+) RNA viruses share some common features during their viral life cycle, e.g., 1) the same viral (+)ssRNA genome serves both as messenger RNA (mRNA) for viral protein translation, and as a viral template for viral duplication, 2) translated viral proteins recruit viral genome to form a viral replication complex (VRC) on intracellular membranes in association with cellular proteins, 3) all (+)ssRNA viruses encode a RdRP for the synthesis of viral RNA template to form a dsRNA intermediate, and 4) special host cell proteins, such as RNA-binding proteins, chaperone proteins, and membrane remodeling or lipid synthesis proteins, are required that collectively participate in the coordination of navigating through intracellular membrane-associated secretory pathways for viral replication 93.

A number of experimental tools could or have been used in yeasts to study life cycle of (+)ssRNA viruses, e.g., 1) an entire viral RNA genome could be transcribed from yeast plasmids and transfected into yeast cells 94,95, 2) both viral RdRP and cellular proteins could be provided in-trans to test for their roles in viral replication 96, and 3) yeast genomic and cDNA libraries as well as the genome-wide gene knock-out libraries were used to study virus-cell interactions 24,28,46,97. By using those sophisticated experimental tools, significant progress has been made in the past several decades to understand the basic aspects of viral life cycle and replication especially those viral steps including translation of viral protein, synthesis of viral genomic template, and association of viral proteins with cellular proteins that are required for viral replication. A number of detailed reviews on these subjects are available 27,45,46,64,74,94. In the following section, specific examples are given in each of those categories.

### Minus-1 ribosomal frameshifting in HIV-1 viral protein translation

The (-1) ribosomal frameshifting is commonly found in many of the RNA viruses including HIV-1 and SARS coronavirus (SARS-CoV). This programed translational frameshifting is a viral mechanism to merge proteins encoded by two overlapping ORFs such as Gag and Pol. The HIV-1 frameshifting site consists of a slippery sequence (U UUU UUA), followed by a stimulatory element P3 98,99. It is part of a larger three-helix structure of the viral RNA genome. The stimulatory element and the slippery sequence pairs with an upstream region to form the second helix. Studies on the HIV-1 frameshifting in the yeast *S. cerevisiae* have contributed significantly to our understanding of this process. In particular, yeast viruses such as the ScV-LA virus also use frameshifting to produce its own viral proteins, a process that is identical to that used by higher retroviruses 92.

A HIV-1 frameshifting yeast model was first established by Wilson and co-workers 100 who produced the Gag-Pol fragment containing the potential frameshifting site of HIV-1 from a yeast expression plasmid. In this way, they were able to monitor the production of the frameshifted protein by western blot analysis. Because of the low efficiency of frameshifting events, initially they failed to identify the stimulatory element. Thus, it was believed that no secondary structure was present in the HIV slippery site 98,100. However, the stimulatory element was later revealed by NMR 101. A follow-up yeast study by using a dual reporter system indeed confirmed a direct correlation between HIV frameshifting efficiency and presence of the stimulatory element as a stem loop 102. Subsequently, it was shown that a number of retroviruses including HIV-1 have the same stimulatory element. Together, studies of slippage efficiencies of the HIV frameshifting site *in vivo* in yeast and *in vitro* in a mammalian system have demonstrated that this process is essential for viral replication and the molecular mechanisms of frameshifting is conserved from yeast to humans 103. Hence, with in-depth understanding of this -1 frameshifting process, it is possible to design specific antiviral drugs by introducing nonsense mutations. For example, alteration of frameshifting frequency or artificial introduction of translational stop by a drug during translational frameshifting could either reduce viral infectivity or halt viral replication 103,104. Therefore, understanding of the -1 ribosomal frameshifting during translation of viral proteins in yeast provided insights into the molecular mechanism of HIV-1 viral replication.

### Initiation of geminivirus replication 

Besides (+) RNA viruses, yeasts have also been used to study viral replication of DNA viruses. They include double stranded DNA (dsDNA) viruses such as human and bovine papillomaviruses (HPVs and BPVs), as well as single stranded DNA (ssDNA) viruses such as Geminiviruses. They all replicate, with various degrees, in yeast (Table 1) 27,29. Geminivirus is the largest *Geminiviridae* family of plant viruses with more than 300 species. Some of the well-studied geminiviruses include African cassava mosaic virus (ACMV), maize streak virus (MSV) and Indian mungbean yellow mosaic virus (IMYMV). These viruses are responsible for significant crop damages worldwide 105. A geminiviral genome consists of either one or two circular ssDNA in the range of 2,500 - 3,100 nucleotides. For the geminivirus with two viral genomic DNA molecules, aka the DNA-A and the DNA-B molecules 106, the DNA-A genome encodes six viral proteins, and the DNA-B produces two movement proteins 107. Although proteins produced by both viral components are involved in viral replication, only the replication-associated protein (Rep) is indispensable for viral replication 108.

Geminiviruses do not have their own DNA polymerases and associated DNA synthesis machinery. They rely on host cellular DNA synthesis machinery to duplicate themselves via a dsDNA intermediate. During the rolling circle replication, Rep serves as a multitasking protein. It involves in viral DNA cleavage and joining after one round of replication. It also has ATPase and helicase activities 109. Since some plant cells are terminally differentiated cells, Rep is responsible for reigniting plant cell cycling by pushing cells from cell cycle G1 phase to S phase where cellular DNA synthesis apparatus is reactivated 110. To achieve this goal, Rep binds to plant homologue of mammalian retinoblastoma protein to promote the G1-S transition 111. In this way, Rep reactivates host cell S phase gene transcription and provides a favorable environment for geminivirus replication 110,112,113.

Studies of geminivirus DNA replication in both yeasts have contributed to our understanding toward the initiation of DNA replication in these groups of higher plant viruses. For example, similar to the role of Rep in plants, the MSV Rep also binds to the maize plant retinoblastoma related-protein (pRbR) protein as it does in plants 114. This allows in-depth functional analysis of the Rep-pRbR interaction in yeast. Indeed, three nucleotide mutations in the MSV Rep-pRbR interaction motif abolished this interaction in yeast and resulted in significant reduction of MSV-induced symptom severity in maize 115. Interestingly, one of the three mutations C(601)A reversed with high frequency in the maize plant, suggesting the functional requirement and selection pressure of the Rep-pRbR interaction during MSV viral replication. Similar to budding yeast, Rep showed very similar activities in fission yeast as it does in plants. For example, ectopic expression of the ACMV Rep alone triggers cellular DNA re-replication in fission yeast 109. Especially, it showed its characteristic DNA cleavage activity, activated DNA synthesis and increased cellular DNA contents without cell division 109. Furthermore, a RXL motif was identified in the Rep protein that might be an alternative link to the Rep-pRbR interaction and cell cycle control 116. Mutation in this motif abrogated Rep-induced DNA re-replication in fission yeast. Consistent with the fission yeast finding, the ACMV containing the same mutation in the Rep motif was unable to induce symptomatic infection in tobacco (*Nicotiana benthamiana*) plants 116.

### Genomic approach to study involvement of cellular proteins in viral replication of tomato bushy stunt virus (TBSV)

Genome-wide approaches have been applied to study viral replication of a number of plant, animal and human viruses. Reviews or reports that cover these topics can be found at 24,28,46,97. For example, TBSV is a (+)ssRNA virus that infects various crops including tomatoes 46,94. The TBSV genome contains five genes that encode a replicase composed of two proteins (p33 and p92), a capsid protein (called CP or p41), a RNA silencing suppressor p19 and a movement protein p22 117. The TBSV replication can be measured in yeast by a three-plasmid TBSV replicon system 95. Two of the plasmids constitutively express the essential TBSV replicase proteins, p33 and p92; the third plasmid drives TBSV replicon’s transcription through an inducible *GAL1* promoter. The TBSV replication is measured by the transcription of the TBSV replicon RNA after induction with galactose, which accumulate at very high level in the wild-type yeast strain 94. A number of genomic and proteomic methods were used to identify yeast cellular proteins that are involved in TBSV replication. For a detailed review of this topic, see 46. In brief, a single gene yeast knock-out (YKO) library was first subjected to a genome-wide screening and revealed 96 genes whose absence either inhibited or stimulated TBSV viral replication 95. Because the YKO library only contains deletions of non-essential genes, additional tests were carried out in a *Tet* promoter-inducible yTHC library, and a temperature-sensitive (*ts*) essential gene library with a total of about 800 genes in each library. Thirty and 101 additional TBSV replication regulators were found, respectively 118,119. By using a reversed approach, a genomic plasmid library containing most of the yeast ORFs (~5,500) were overproduced in a TBSV replicon-containing yeast strain. A total of 141 proteins were identified. Among them, 40 proteins increased and the remainder decreased the accumulation of TBSV replicon RNA 119. A total of 36 overlapping yeast proteins were identified based on previous screens of other viruses. A conserved protein kinase C1 (Pkc1) that was shown to inhibit TBSV replication was chosen to validate their results. Indeed, a *ts* mutant of Pkc1 led to a high level of TBSV replication, supporting the idea that Pkc1p is an inhibitor of TBSV RNA replication. Consistently, a specific Pkc1p inhibitor, cercosporamide, also resulted in increased TBSV replication in yeast, plant cells, and in whole plants, confirming that Pkc1 and its associated pathways are involved in regulation of TBSV replication 119.

## YEAST FOR UNDERSTANDING VIRUS-HOST INTERACTIONS

### Virus-mediated cell cycle regulation

Viruses typically encode a limited number of proteins. They have to rely on host cellular resources to complete their life cycle. Thus, they will take a variety of devious approaches to create a cellular environment for the benefit of their own reproduction 120. One common viral strategy is to subvert host cell cycle into a specific phase of the cell cycle where the virus gains maximal benefit. For example, many dsDNA viruses such as HPV and SV40 infect quiescent cells. After infection, they drive cells to S phase of the cell cycle where the pool of deoxynucleotides is high, thus providing an environment that is conducive to viral DNA synthesis 121. A similar viral action was also noted in ssDNA viruses such as geminiviruses that drive cell cycle G1-S transition 110,112,113. Other viruses such as HIV-1 and herpes simplex viruses (HSV) induce cell cycle arrest. The possible objective is to avoid competition of cellular resources between the virus and the normal host cellular metabolism 122. Additional benefits to virus-induced cell cycle arrest could include avoiding host antiviral immune responses, maximizing availability of the cellular resources for its transcription, translation and assembly, and delaying programmed cell death until completion of the viral replication 123,124,125.

Other viral proteins that regulate cell cycle in yeasts include SV40 RF-S 126, HIV-1 viral protein R (Vpr) 127,128, HTLV-1 Tax 129, HPV E2 130, TBSV Rep 112, and ZIKV proteins 36. In the followings, HIV-1 Vpr is used as an example to illustrate how fission yeast was used to delineate molecular mechanism of Vpr-induced cell cycle G2 arrest. For extensive reviews of this topic, see 63,64,72. Note that expression of HIV-1 *vpr* gene in budding yeast resulted in cell growth arrest. It, however, did not induce cell cycle G2 arrest as it was shown in mammalian and fission yeast cells 127,131,132.

HIV-1 Vpr is a virion-associated viral protein of about 12.7 kD. Its function is required both *in vitro* and *in vivo* for efficient viral infection of non-dividing mammalian cells such as monocytes and macrophages 133,134,135. It is a multifaceted protein that is involved in multiple steps of the HIV-1 life cycle 136. It involves in cytoplasmic-nuclear transport of proviral integration complex (PIC), activates HIV-1 LTR (long terminal repeat) promoter for viral transcription, and induces cell death through apoptosis 136. In addition, it induces cell cycle G2 arrest in both human and fission yeast cells, suggesting a highly conserved activity of this viral protein 128,134,137. Vpr-induced cell cycle G2 arrest in host CD4(+) T-cells was thought to avoid host immune response 138. It was also shown that HIV-1 in cells that arrested in the G2 phase of the cell cycle by Vpr replicates at its maximum level 139.

Cell cycle G2-M transition is a tightly regulated cellular process that requires activation of the Cdc2 kinase (a human homologue of CDK1), which determines onset of mitosis in all eukaryotic cells. In both human and fission yeast cells, the activity of Cdc2/CDK1 is regulated in part by the phosphorylation status of tyrosine 15 (Tyr15) on Cdc2/CDK1, which is phosphorylated by Wee1 kinase during late G2 and is rapidly dephosphorylated by the Cdc25 tyrosine phosphatase to trigger cellular entry into mitosis. These mitotic Cdc2/CDK1 regulators are safeguarded by two well-characterized mitotic checkpoint pathways to prevent cells from entering mitosis when cellular DNA is either damaged (the DNA damage G2 checkpoint pathway) or when DNA replication is compromised (the DNA replication checkpoint pathway) 63,140,141.

HIV-1 Vpr induces cell cycle G2 arrest by subverting the same cell cycle G2-M regulatory apparatus as described above. Specifically, it promotes hyper-phosphorylation of Tyr15 of Cdc2/CDK1 in mammalian and fission yeast cells 128,142. The inhibitory effect on Cdc2/CDK1 is achieved by inhibition of the Cdc25 phosphatase and activation of the Wee1 kinase 143,144,145. Subsequent studies in mammalian cells showed that Vpr-induced G2 arrest is mediated through direct binding of Vpr with a Vpr-binding protein (VprBP) 146,147, which is part of the ubiquitin E3 enzyme, suggesting possible involvement of the ubiquitin proteasome system. Indeed, Vpr associates with the proteasome both in fission yeast and mammalian cells 148. Specifically, in fission yeast, it associates with the 19S subunit of the proteasome through 19S-associated Mts4 and Mts2 proteins. This Vpr-19S proteasome interaction was further confirmed in mammalian cells where Vpr associates with the same two mammalian orthologues (Mts4 and S5a) of the fission yeast proteins 148. In addition, Rhp23, a fission yeast homologue of human DNA excision repair protein hHR23A and budding yeast RAD23, was shown to be critical for Vpr-proteasome interaction and was involved in the Vpr action 149,150. Interestingly, even though both ATM and ATR were shown participating in Vpr-induced G2 arrest, implicating involvement of mitotic DNA damage or DNA replication checkpoint pathways 151,152, neither of these two classic mitotic checkpoint control pathways was exclusively responsible for the G2 arrest induced by Vpr. Further fission yeast and mammalian studies showed that Vpr induces G2 arrest via a protein phosphatase 2A (PP2A)-mediated cellular pathway 145,153,154. Unlike the conventional cell cycle G2-M regulation, Vpr also induces cell cycle G2 arrest at least in part through a mechanism involving in a fission yeast kinase Srk1 and its human homologue MK2 69. These results suggest that Vpr might modulate cell cycle G2 transition through an alternative and possibly a novel cellular mechanism other than the classic mitotic checkpoints 63,72,155. Indeed, a later study showed that Vpr induces cell cycle G2 arrest through a unique molecular mechanism that regulates host cell cycle regulation in an S-phase dependent fashion 156. Altogether, this example demonstrates that fission yeast can indeed be used as a reliable model organism to dissect molecular mechanism of HIV-1 Vpr-induced cell cycle G2 arrest. It was the combined results generated from the fission yeast model system with the study and verification in mammalian cells that led to the finding that Vpr induces cell cycle G2 arrest through a unique virus-mediated cellular mechanism.

### Virus-mediated cell death and apoptosis

Viral infection could cause cell death through at least three different ways in yeasts and higher eukaryotes, i.e., necrosis, apoptosis or autophagy-mediated cell death 157. Necrosis is a form of cell death that is caused by factors external to the cell such as viral infection, which results in the unregulated digestion of cell components. In contrast, apoptosis is a naturally occurring and programmed process of cellular death. Autophagy is a normal cellular process that maintains cellular homeostasis. It regulates protein degradation and turnover of the destroyed cell organelles. In response to cellular stress such as nutrient starvation or viral infection, autophagy is activated. However, prolonged activation of autophagy often results in autophagy-mediated cell death by either cell necrosis or apoptosis. Thus, autophagy and cell death are regulated balance of two cellular events.

The processes of yeast cell death resemble in many ways those of higher eukaryotes 158,159,160,161. Thus, yeast could serve as model organism to study these terminal cellular processes. For example, yeast has been used as a model to study yeast necrosis. In contrary to the traditional belief that necrosis is normally a passive cell dying process, evidence accumulated over more than a decade suggests there is actually a regulated necrotic program that controls how long a cell will live (longevity) or die 16. As for yeast apoptosis, there was long skepticism as whether yeast has true apoptosis. However, this cynicism starts to dissipate by the increasing evidence generated from yeast studies in the past two decades. In particular, like in mammalian cells, yeast apoptosis is also mediated through a caspase-mediated proteolytic process in addition to other characteristic apoptotic features 162,163,164. In fact, some of the same mammalian pro-apoptotic or anti-apoptotic regulators were found in yeasts that show similar activities to higher eukaryotes. For detailed reviews of this subject, see 158,161,165,166,167,168. Although yeast apoptosis is not as well studied in fission yeast as in budding yeast, an apoptotic-like process does seem to be present in fission yeast 169. For instance, expression of mammalian pro-apoptotic proteins Bax and Bak induce apoptosis-like cell death that was strongly suppressed by co-expression of the anti-apoptotic protein Bcl-XL 170,171. A pombe caspase 1 (Pca1) was identified and its budding yeast homologue Yca1p was shown to be a *bona fide* caspase 172. Moreover, both caspase-dependent and -independent processes are present in fission yeast 172,173. Therefore, at least some of the mammalian apoptotic processes are present in yeasts.

Yeast killer strains that carry (+)dsRNA viruses or produce viral toxins induce yeast apoptosis in sensitive or targeted non-infected yeast cells 89. Such yeast-mediated apoptosis typically occurs at low-to-moderate concentration of viral toxins in those cells; whereas necrotic cell death takes place at high concentration, suggesting activation of apoptotic or necrotic cellular death regulators requires different thresholds of stimuli. The viral toxins such as the pore-forming proteins, K1, K2, and zygocin, kill yeast cells by disrupting the cytoplasmic membrane; the protein toxins such as K28 induce cell cycle G1/S cell cycle arrest and thereby block DNA synthesis in the nucleus 89.

Expression of exogenous viral proteins also induce cell death and apoptosis in yeasts 64,174. Viral proteins that induced yeast cell death and apoptosis include, but are not limited to, pro-apoptotic proteins such as adenovirus E4orf4 protein 175, HIV-1 Vpr 67,176, HIV-1 protease (PR) 177,178,179,180, and ZIKV proteins 36. Anti-apoptotic viral proteins include baculovirus p35 174 and DPV022 protein of the Deerpox virus 181. In the followings, we present two HIV-1 viral proteins (Vpr and PR) as examples to demonstrate how studies on virus-mediated yeast cell death and apoptosis were carried out in budding and fission yeast.

HIV-1 PR is an essential viral enzyme. Its primary function is to proteolyze the viral Gag-Pol polyprotein for production of viral enzymes and structural proteins as well as for maturation of infectious viral particles. HIV-1 PR induces cell death/apoptosis presumably due to its ability to proteolyze vital host cellular proteins 177,182,183,184. The coupling between PR-induced cell death/apoptosis and proteolysis was demonstrated by the fact that HIV-1 PR inhibitors (PIs) prevent PR-induced cell death/apoptosis 183,184,185,186. HIV-1 PR induces apoptosis in mammalian cells by caspase-3 cleavage and interruption of mitochondrial functions 178,187. Similar to its cell death/apoptotic effect in mammalian cells, HIV-1 PR also induces cell death in both budding and fission yeast 35,177. Those cell killing effects were caused by HIV-1 PR proteolytic activities because HIV-1 PIs also suppressed PR-induced cell killing in both yeasts 179,180,188. Interestingly, however, HIV-1 PR kills budding yeast resulting in cell lysis; whereas no cell lysis was observed in fission yeast. The difference between these two yeasts could potentially be explained, at least in part, by the relative thicker cell wall of fission yeast than budding yeast 179,180. Studies in the fission yeast further demonstrated that HIV-1 PR cleaves its indigenous viral protein target sequences such as matrix and p6 proteins 189,190. Moreover, PR-induced cell death triggered the reactive oxidative species (ROS) production, an indication of oxidative stress. It also caused changes in mitochondrial morphology that are linked to apoptosis 67,191. Together, those data suggested that HIV-1 PR displays the same enzymatic activity as it does during HIV-1 infection of mammalian cells. In order to explore the molecular interactions of HIV-1 PR with cellular proteins, a genome-wide screen was launched to search multicopy HIV-1 PR suppressors using a fission yeast genomic cDNA library in HIV-1 PR-producing fission yeast cells. A fission yeast serine/threonine kinase (Hhp2) was identified as a novel PR suppressor that suppresses HIV-1 PR-induced PR protein cleavage and cell death in fission yeast 179. Significantly, Hhp2 kinase suppressed, at least in part, HIV-1 PR-induced cell death and apoptosis in mammalian cells 179.

Vpr also induces cell death in budding and fission yeast 128,192. HIV-1 Vpr-induced cell death/apoptosis contributes to the depletion of CD4 T-cells in HIV-infected patients 193. Further characterization of Vpr-induced cell death in budding yeast showed that the C-terminal domain of Vpr is primarily responsible for the cell killing effect in yeast and mammalian cells 194,195. When the C-terminal Vpr was subject to intact mammalian cells or purified mitochondria, it induced apoptosis through a permeability transition pore complex (PTPC) of mitochondria 176. Consistently, yeast strains lacking part of the PTPC showed reduced Vpr-induced killing than the wildtype control cells.

Similar to Vpr-induced apoptosis in mammalian cells, Vpr triggers ROS production, promotes phosphatidylserine externalization and induces hyperpolarization of mitochondria in fission yeast, leading to changes of mitochondrial membrane potential 67. These data suggested that HIV-1 Vpr-induced cell death in fission yeast is reminiscent of apoptosis. To further explore Vpr-fission yeast cell interaction during Vpr-induced cell death/apoptosis, a genome-wide functional search for multicopy protein suppressors of Vpr-induced cell death/apoptosis was conducted by overproducing a fission yeast cDNA library in a Vpr-producing fission yeast strain. A novel anti-apoptotic protein, translational elongation factor 2 (EF2) was isolated 66. It not only suppresses Vpr-induced cell death in fission yeast but it also suppresses Vpr-induced apoptosis in mammalian cells through caspase 9 and caspase 3-mediated mechanism 66,67.

### Functional characterization of small viral genomes in fission yeast

**Figure 2:**
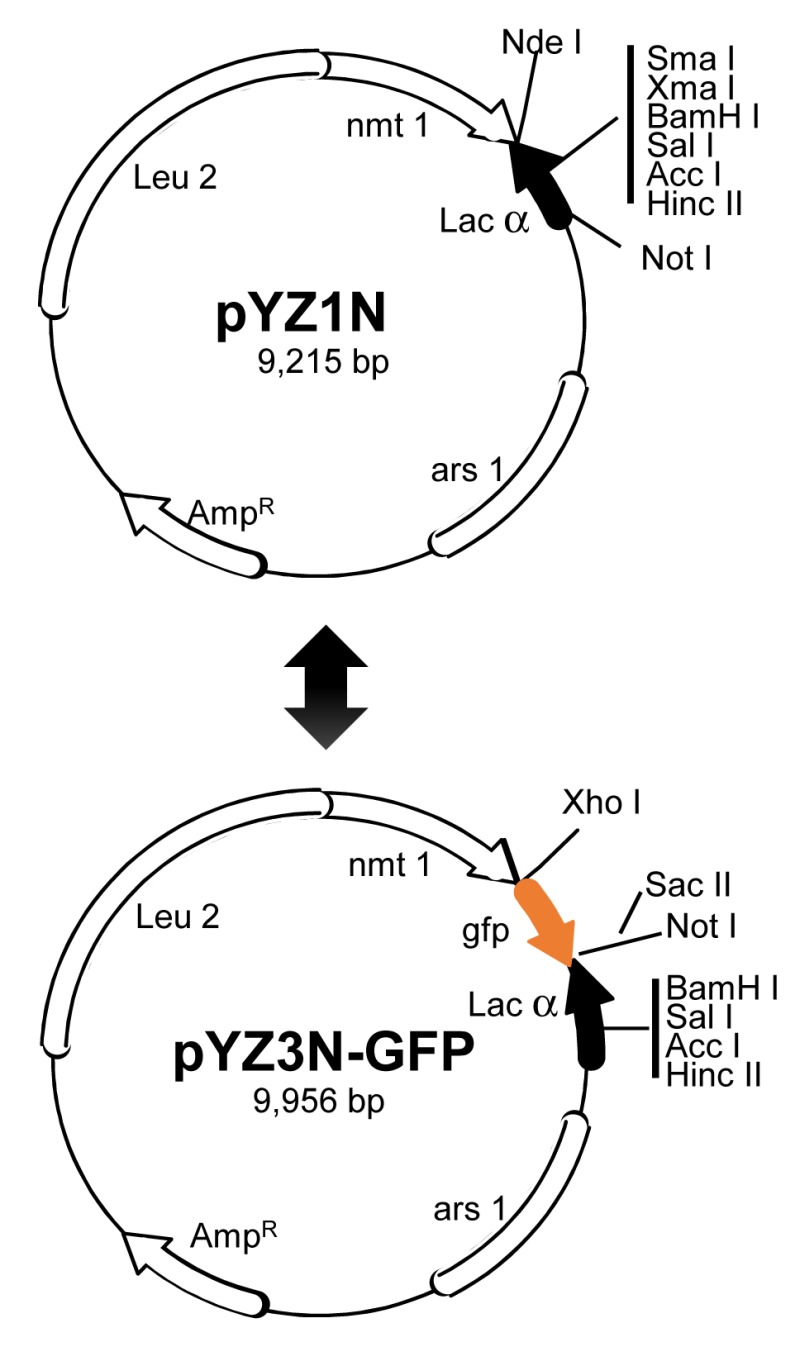
FIGURE 2: Schematic diagram of a shotgun approach to clone a small viral genome in fission yeast. **(Top) **the pYZ1N vector contains a wild type *nmt1* promoter 68,196. It carries a *LEU2* gene for selection. pYZ2N contains the wild type *nmt1* promoter and a *URA4* gene selection marker. **(Bottom)** pYZ3N-GFP contains the same *nmt1* promoter as pYZ1N and has an added green fluorescent protein GFP tag. The α-peptide of β-galactosidase is used for selecting DNA inserts with α-complementation. Unique cloning sites in these vectors are indicated. *ars1*, origin of replication from *S. pombe*; Leu2, *Saccharomyces cerevisiae* leucine biosynthesis gene; AmpR, bacterial ampicillin resistance gene. Adapted from 36,68. Note that nothing is new in molecular features of shuttle vectors described. Goal of this figure is to illustrate a robust and streamlined strategy of shotgun gene cloning of a small viral genome.

As described in the previous sections, yeasts have proven to be fruitful hosts to conduct genome-wide studies on virus-host interactions, particularly because their small genomes and genetic amenability. For the same token, yeast could also, in principle, serve as a surrogate to carry out functional study of small viral genomes. It is conceivable that effects of single or multiple viral gene products could be tested separately or simultaneously in the same yeast strain, thus allowing testing the same basic cellular function that is affected by individual or combination of different viral proteins. Besides all of the operational and genetic advantages of using yeast as a model organism, a large-scale gene cloning strategy and a streamlined functional characterization system are also needed for this purpose. Example of such a fission yeast system is shown in Fig. 2 68,70. Please note that there is nothing new in the molecular features of these shuttle vectors. Goal of Fig. 2 is to illustrate the combined use of these vectors will provide a robust and streamlined shotgun strategy of a small viral genome. Thus, notable features of this fission yeast system include 1) the gene cloning process is streamlined to a sequential order to add or remove the green fluorescent protein (GFP) tag. For example, molecular cloning of a viral gene into one of the pYZ3N-GFP-carrying vectors generates a 5’ GFP-tagged viral protein that can be used for the determination of its subcellular localization; the pYZ1N gene expression vector and its derivatives are used for functional characterization of a viral protein without the GFP tag; 2) all of the gene cloning is done in an unidirectional fashion with positive identification of the gene insertions, based on α-complementation of X-gal in *Escherichia coli*; 3) an inducible gene transcriptional *no message in the thiamine* (*nmt1*) promoter 68,196 is used to allow measurement of the viral gene-specific effect; 4) three different strengths of the *nmt1* promoter (high, intermediate and low) with two different cell growth selection markers (*leu2* and *ura4*) 68,196 allow testing of gene expression at various levels or testing of viral protein-to-protein interactions 68,196; and 5) multiple viral gene-producing yeast strains can be established and maintained that allow simultaneous viral gene testing , thus facilitating functional characterization of a small viral genome. By using this system, functions of small viral genomes such as HIV-1 or ZIKV have been characterized as outlined below 35,36.

The HIV-1 RNA genome is about 9 kb that encode a total of nine ORFs including Gag, Pol and Env polyproteins, four accessory proteins (Vpu, Vif, Vpr, Nef) and two regulatory proteins (Rev, Tat). In a genome-wide and functional analysis of the HIV-1 genome in fission yeast, each one of the HIV-1 genes was cloned and expressed individually in a wild type fission yeast strain 35. The effect of protein expression on basic cellular functions such as subcellular location, cell proliferation, and cytotoxicity were observed. Overall, there is a general correlation of subcellular localization of each viral protein between fission yeast and mammalian cells. Three viral proteins, Vpr, PR and Rev, affected various cellular activities. Only the Rev effect is described below as the effects of HIV-1 Vpr and PR have already been described in the early sections.

HIV-1 Rev is an 18 kD phosphoprotein with 116 amino acids that contains a nuclear localization signal (NLS) and nuclear export signal (NES) 197,198. It mediates nuclear export of partially spliced and un-spliced viral transcripts through its NES allowing nucleocytoplasmic shuttling, thus playing a pivotal role in viral replication 199,200. It interacts with a small nucleoporin-like protein hRIP/RAB1 and yRip1p in mammalian and budding yeast, respectively 201,202. Consistent with the role of Rev in nucleocytoplasmic shuttling, Rev localizes predominantly in the fission yeast nucleus with minor presence in the cytoplasm 35. In addition, production of HIV-1 Rev protein appeared to reduce cellular growth in fission yeast and mammalian cells, but this sluggish cellular growth did not lead to cell death. Paradoxically, however, Rev causes death in non-dividing human cells 200. Further tests revealed that Rev triggered transient ROS production. This result suggested that the fission yeast cells were likely experiencing transient intracellular oxidative stress. Typically, constant induction of oxidative stress should produce large and persistent amounts of ROS that should have caused cell death 203,204. Thus, the observed differences in patterns of ROS production could potentially explain why Rev induces cell death in non-dividing human cells but not in proliferating yeast cells 200.

Another and the most recent example of using fission yeast to carry out large-scale molecular cloning and functional characterization of a small viral genome is the study of the ZIKV 36. ZIKV was thought to be a mild virus that had limited threat to human health. However, the most recent ZIKV outbreak in the Americas surprised us because of its rapid global spread and the discovery that ZIKV causes severe neurologic disorders or birth defects including microcephaly and the Guillain-Barré syndrome 205,206,207,208. So the question is why the ZIKV suddenly has become so virulent in humans? ZIKV infection studies in human brain and neural progenitor cells suggested that ZIKV conferred various cytopathic effects (CPEs) including reduced neural cell proliferation, cell cycle dysfunction and increased cell death/apoptosis 209,210. Those CPEs correlated with the neuronal cell-layer volume of the brain organoids resembling microcephaly, suggesting ZIKV-induced microcephaly is high likely the result of ZIKV-mediated increase of CPEs 205,206,207,211. The next and logic question is which ZIKV viral protein(s) is responsible for the observed increase of CPEs? To address this question in a timely manner, fission yeast was used as a surrogate to embark on a rapid genome-wide analysis of ZIKV proteins 36. Fission yeast is particularly useful here because those ZIKV-mediated CPEs are high likely conserved cellular activities 2,6,212.

ZIKV is a small (+)ssRNA virus that has a viral genome of approximately 10.7 kb 213. The ZIKV genome encodes a single polyprotein that is cleaved by viral and host proteases into 14 proteins and small peptides, i.e., six structural (anaC→C, PrM→M, Pr and E), seven nonstructural (NS1, NS2A, NS2B, NS3, NS4A, NS4B and NS5) proteins and a small peptide 2K 36,214. Each one of the 14 ZIKV viral cDNA that encodes a specific protein product was cloned into the fission yeast gene expression systems (Fig. 2) 68. All of the ZIKV viral activities were measured simultaneously under the same inducible conditions thus it expedited the functional characterization of the ZIKV genome. Consistent with the notion that ZIKV is a cellular membrane-associated virus and ER is the major ”viral factory“ 215,216,217, 9 out of the 14 ZIKV proteins associated with the ER network, including nuclear membrane, ER to Golgi apparatus 215,217,218. Seven ZIKV proteins (five structural proteins and two non-structural proteins) conferred some of the same CPEs as reported in the ZIKV-infected mammalian cells 36,205,209,211,215. Specifically, they also restricted cellular growth, triggered cellular autophagy, caused cell cycle dysfunction and cell death 36. Consistently, some of the same ZIKV protein effects such as NS4A have already been reported in human cells 219. For example, NS4A triggers autophagy in human fetal neural stem cells through inhibition of the mammalian TOR pathway via AKT 219. In fission yeast, five ZIKV proteins including NS4A triggered autophagy as indicated by the formation of yeast cytoplasmic puncta 36,220. Further analysis showed that NS4A activates autophagy through the cellular TOR stress pathway that connects NS4A-mediated oxidative stress and the ROS production. Fission yeast study further showed that the inhibitory NS4A effect on TOR was mediated through Tor1 and Tip41, which are the human equivalents of TSC1 and TIP41 proteins 221,222. Altogether, these yeast findings provide a foundation for future research of viral cytopathic factors that contribute to the increase of viral pathogenicity and possibly the induction of microcephaly 223.

## YEAST FOR DRUG DISOCVERY

### Yeast as a cell-based high-throughput system for the discovery of small molecule antiviral drugs

Small molecule antiviral drugs could be very effective as in treating e.g, HCV or HIV infections. In these two cases, the antivirals could cure HCV infection or eliminate the HIV virus to non-detectable levels 224,225. However, currently, there are very limited number of antiviral drugs on the market to fight for other major and clinically important viral infections such as influenza, hepatitis B virus (HBV) and ZIKV. Moreover, emerging drug resistance is also a major concern 180,226,227,228. Therefore, there are urgent and constant needs to develop new and better antiviral drugs.

Research and development of a new US FDA-approved drug takes on average of at least ten years with a total cost of more than one billion of US dollars 229. Even with such a long time period and high cost, the average success rate of a lead drug candidate that reaches to its final approval is approximately one out of ten thousands 230. Majority of the lead compounds failed because of the drug cytotoxicity or drug associated adverse side effect. Hence, it is always desirable to develop a fast, large-scale and cost-effective drug discovery process that, at the end, could generate a drug that is target-specific and less toxic.

Cell-based and high throughput drug screening (HTS) systems could be used to for the new drug discovery. The advantages of a cell-based assay include 1) cytotoxic compounds are automatedly removed from the HTS drug screenings, 2) the drug screening could be designed against a heterologous gene target, thus it is target-specific, and 3) unlike the structure-based drug designs, cell-based drug screening is functionally driven. Thus, it has the potential to identify novel inhibitors such as allosteric inhibitors, i.e., an inhibitor that inhibits the viral target activity regardless of whether it binds to the target as the structure-based design does. Yeasts offer additional advantages over the use of mammalian cell systems. For example, yeast cells grow much faster than mammalian cells and they are very easy to maintain in a large-scale setting. Thus, it is more cost-effective than using mammalian cells. Yeasts are also genetically amendable for stable gene expression by integrating the viral gene of interest into yeast chromosomes. Inducible expression of the target gene-of-interest further allows target-specific drug screening. Another and important benefit is that yeast is non-infectious to humans. Altogether, yeasts offer a rapid, cost-effective and non-biohazardous HTS system for the discovery of non-cytotoxic, target-specific and possible novel class of antiviral drugs.

Both fission and budding yeast have been used to develop HTS for the discovery of antiviral drugs, which include HIV-1 10,231,232, CMV 233, Epstein-Barr virus 234, SARS-CoV 235 and influenza virus 236. For example, CMV PR is an essential viral enzyme for viral replication. Inhibition of this vital enzyme by CMV inhibitors suppresses CMV viral replication 233,237. A budding yeast cell-based system was developed by inserting the CMV PR cleavage sequence into the yeast Trp1p isomerase gene 234. Inactivation of the Trp1p by CMV PR-mediated cleavage of Trp1p causes cell growth arrest. Thus, when the CMV PR activity is inhibited by a CMV PR inhibitor such as BI31 or BI36, yeast cells restore normal cellular growth 234.

Another budding yeast cell-based HTS assay was for the Matrix-2 (M2) proton channel protein of the influenza A virus 236. The M2 proton channel is a homotetramer that is an integral part of the viral envelope, hence it is essential for viral replication. A similar growth restoration assay was developed as expression of M2 inhibits yeast cell growth. Thus, the M2 proton channel was used as an anti-influenza drug target. HTS screening of 150,000 compounds yielded 21 anti-M2 inhibitors including the known M2 inhibitors of amantadine and rimantadine 238.

Fission yeast has also been used for the development of antiviral HTS against HIV-1 Vpr and PRs 179,180,232. In the case of HIV-1 Vpr, its activities are associated with increase of viral replication and depletion of CD4 T-lymphocytes, a hallmark of HIV-1 infection. Slow disease progression with low viral load has also been linked to Vpr-defective viral infections in rhesus monkeys, chimpanzees or HIV-infected patients 239,240,241,242,243, suggesting Vpr could potentially be used as a new drug target for anti-HIV therapies. In addition, Vpr prevents cell proliferation, induces cell cycle G2/M arrest and causes cell death both in fission yeast and mammalian cells 63,64,244, thus providing separate and unique cellular endpoints to set up the primary, second and counter-screen assays that are typically required for the HTS drug screening. Since Vpr blocks cell growth and induces cell death and apoptosis, an absorbance-based assay measuring Vpr-induced growth arrest was used as the primary assay. Automated fluorescent detection of Vpr-induced cell death by the yeast live/dead assay was used as a secondary assay. Production of GFP driven by the same *nmt1* promoter as the primary and secondary assays was used as a counter-screen assay to eliminate potential false positives due to the inhibitory activity against the *nmt1*-mediated transcription 232. This HTS platform was used to screen against more than 400,000 of small molecule compounds at the National Chemical Genomic Center of the National Institute of Health, USA. A total of 165 lead compounds were found to have various levels of inhibitory activities against Vpr. Among them, three clusters of chemical compounds were identified.

A fission yeast cell-based assay was also developed for HIV-1 PR 35,179. HIV-1 PR is a major therapeutic target in antiretroviral therapies (ARTs) because it is an essential viral enzyme 231. Indeed, HIV-1 PI is currently the most potent class of anti-HIV drugs. Monotherapy with PI alone can reduce HIV-1 viral load by several logs 245. Besides HIV-1 PR-induced yeast cell death, HIV-1 PR also functions as a proteolytic enzyme in fission yeast in the same manner as it does in mammalian cells 35,177. It cleaves the same indigenous HIV-1 viral p6/MA protein substrate as it does during natural HIV-1 infections. Moreover, both PR-induced cell death and proteolytic cleavages can be prevented by PR-specific enzymatic inhibitors, Indinavir (IDV), Darunavir (DRV) and other PIs 179,180. Most interestingly, multi-PI resistant HIV-1 PRs, which were isolated directly from HIV-1 infected patients, also showed the same viral activities in fission yeast. In addition, those multi-PI resistant PRs retained the same drug resistant profiles in the fission yeast as they do in mammalian cells 180. This opens up a unique opportunity to use fission yeast as surrogate system to develop HTS systems against HIV-1 superbugs that are resistant to many, if not all, of the existing protease inhibiting drugs 180,226,227.

Note that fission yeast has a thick cell wall. Thus, there certainly are differences in cell membrane permeability and drug uptakes between yeast and human cells. Generally, much higher drug concentration than that used in mammalian cells is required in fission yeast to achieve the same inhibitory effect of a viral target. However, this should not be a functional concern because HIV-1 PIs such as IDV, DRV and others effectively inhibited the same HIV-1 PRs in a dose-dependent manner as they do in mammalian cells 179,180. Nevertheless, the effective inhibitory concentration of an inhibitory compound identified from yeast must be re-calibrated when it is used in testing of mammalian cells.

## CONCLUDING REMARKS

Both budding and fission yeast have been used as model systems for the study of plants, animal and human viruses in the past century. There is no doubt that, through those yeast studies, significant progress has been made toward our understanding of those viruses and their interactions with cellular proteins. However, we should also be mindful that, after all, yeasts are not plants, animals or humans. Any new findings from the yeast models must be verified in their respective hosts. Therefore, it is important to know the limitation of yeasts as they may not be suitable to study every aspect of the virus. For example, budding yeast should not be the preferred choice to study mRNA processing or siRNA because only a small percentage of yeast protein-coding genes contain introns, nor does it have a comparable siRNA process with higher eukaryotes. Similarly, fission yeast should not be used to study peroxisomes. However, both yeasts are well-suited to study cell cycle regulation and some aspects of the programed cell death. It is also worthwhile mentioning again that the use of yeasts as a model tool saves cost, and provides unique tools to identify highly conserved cellular factors that interact with the virus of interest. In particular, when the use of yeast to study virus is combined with the tools of higher eukaryotic biology and virology, it will empower us with a unique set of tools. Such a distinctive combination of tools could give rise to unique perspectives of scientific findings that are otherwise difficult to obtain based solely on a single approach or organism.
